# Evidence of TCM Theory in Treating the Same Disease with Different Methods: Treatment of Pneumonia with *Ephedra sinica* and *Scutellariae Radix* as an Example

**DOI:** 10.1155/2020/8873371

**Published:** 2020-11-28

**Authors:** Liping Sun, Dandan Wang, Yan Xu, Wenxiu Qi, Yanbo Wang

**Affiliations:** ^1^Changchun University of Chinese Medicine, Changchun 130117, China; ^2^Affiliated Hospital of Changchun University of Chinese Medicine, Changchun, Jilin 130021, China

## Abstract

Pneumonia is a serious global health problem and the leading cause of mortality in children. Antibiotics are the main treatment for bacterial pneumonia, but there are serious drug resistance problems. Traditional Chinese medicine (TCM) has been used to treat diseases for thousands of years and has a unique theory. This article takes the treatment of pneumonia with *Ephedra sinica* as a representative hot medicine and *Scutellariae Radix* as a representative cold medicine as an example. We explore and explain the theory of treating the same disease with different TCM treatments. Using transcriptomics and network pharmacology methods, GO, KEGG enrichment, and PPI network construction were carried out, demonstrating that *Ephedra sinica* plays a therapeutic role through the NF-*κ*B and apoptosis signaling pathways targeting PLAU, CD40LG, BLC2L1, CASP7, and CXCL8. The targets of *Scutellariae Radix* through the IL-17 signaling pathway are MMP9, CXCL8, and MAPK14. Molecular docking technology was also used to verify the results. In short, our results provide evidence for the theory of treating the same disease with different treatments, and we also discuss future directions for traditional Chinese medicine.

## 1. Background

Pneumonia is a serious global health problem [1] and the leading cause of death in children [2]; pneumonia can be caused by bacteria, viruses, fungi, and other microorganisms [3]. Antibiotics are currently the most important treatment for bacterial pneumonia and have been used for a variety of bacterial diseases. The Global Antimicrobial Resistance Surveillance System (GLASS) report released by the World Health Organization in 2019 reveals a worrying reality: the emergence and spread of antibiotic resistance worldwide has directly led to prolonged disease treatment cycles, increased complications, and even death [4, 5]. Therefore, there is an extremely urgent need to explore new methods to treat pneumonia. Traditional Chinese medicine, the most popular traditional medicine system in the world, is the traditional medicine system in China and has been guided by materialistic philosophy for thousands of years. We think that exploring new ways to use traditional Chinese medicine to treat diseases will help to improve modern medicine and support the development of new drugs.

**Figure 1:**
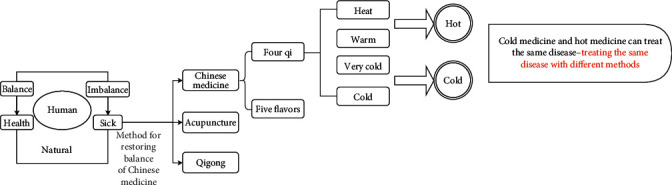
Disease development and treatment in Chinese medicine.

Traditional Chinese medicine (TCM) is different from modern medicine and has a unique system of preventing, diagnosing, and treating diseases based on the philosophy of naive materialism [6]. The core idea is the equilibrium state between humans and nature. Theories including Yin and Yang and the Five Elements are intended to explain this equilibrium state. When people and nature are out of balance, diseases will occur. At this time, methods such as Chinese medicine, acupuncture, tai chi, and qigong can be used to correct this imbalance [7, 8], as shown in Figure 1. Among these methods, Chinese medicine has the characteristics of four kinds of “qi.” The four qi are the four natural attributes of being very cold, cold, hot, and warm. Hot and warm are distinguished by the degree of heat, and very cold and cold are distinguished by the degree of coldness. These types of qi are used to correct different degrees of heat and coldness in the human body [9]. Most of the time, the theory of TCM has explored the method of harmonizing people with nature from the perspective of a philosophical theory. Although Chinese people take pride in the theory of TCM, there is no modern scientific evidence for its effectiveness.

Since the launch of the Human Genome Project in the 1990s, large-scale research on genes and their expression products has emerged, such as genomics, transcriptomics, and proteomics, and related technologies have developed rapidly. Among them, transcriptomics can systematically detect the cell status and comprehensively reflect the real-time expression status and degree of degradation, which is extremely important for the research of human diseases. There have been many omics studies of TCM before. For example, Wu et al. studied the application of metabolomics in the treatment of coronary heart disease with TCM [10]. Zhao et al. also used metabolomics techniques to explore the anti-inflammatory effects of Zhishi and Zhiqiao [11]. Peng et al. integrated transcriptomics, proteomics, metabolomics, and systems pharmacology to explore the mechanism of the Bufei Yishen formula in treating chronic obstructive pulmonary disease [12]. However, research using omics technology to explain the theory of TCM is very rare. Only one study has been reported, in which He et al. used transcriptomics technology to discuss the blood stasis theory of TCM [13]. Many theories of Chinese medicine are the soul of Chinese medicine and the basis for guiding clinical medication. Therefore, the use of omics technology to explore TCM theory is both innovative and meaningful research.

**Figure 2:**
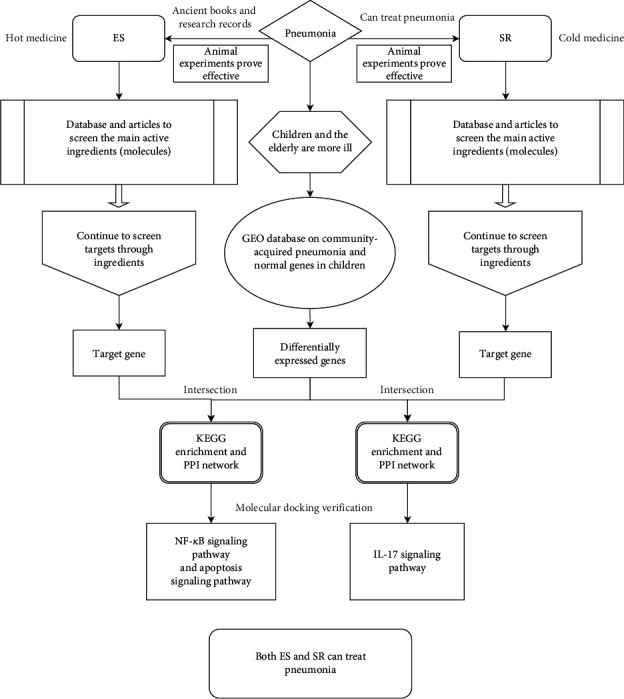
Research schematic.

In TCM theory, both cold and hot medicines can be used to treat the same disease, which is called “treating the same disease with different treatments” [14], which means that different treatment methods are used at different stages of disease development [15]. Based on the number of studies and citations in the published literature, we selected *Ephedra sinica* (ES), a small shrub in the genus *Ephedra*, which has the function of dissipating wind-cold, freeing the lungs, and relieving asthma and is considered warm in Chinese medicine, as a representative of warm drugs. *Scutellariae Radix* (SR), the dry root of the Labiatae plant *Scutellaria baicalensis* Georgi, which has the functions of clearing heat and dampness, purging fire, and detoxifying and is considered to be cold, was chosen as a representative of cold drugs. Both drugs are believed to have therapeutic effects on pneumonia. Briefly, the aim of this study was to investigate the specific mechanisms of ES and SR, two representative Chinese medicines for the treatment of pneumonia, using network pharmacology and transcriptomics combined with animal experiments. To clarify the four types of qi in Chinese medicine and guide clinical treatment, a research schematic is shown in Figure 2. Our results suggest that ES and SR treat bacterial pneumonia via different signaling pathways and confirm the theory of treating the same disease with different methods in TCM.

## 2. Methods

### 2.1. Molecular Information and Target Sets for ES and SR

The molecular information of ES and SR was obtained from the TCMSP database (http://tcmspw.com/tcmsp.php) constructed by previous researchers, and their physical and chemical properties, such as molecular weight (MW), the number of donor atoms for H-bonds (nHDon), the number of acceptor atoms for H-bonds (nHAcc), and the Moriguchi octanol–water partition coeff. (log P), were analyzed [16]. To further explain the absorption, distribution, metabolism, and excretion (ADME) process of these two drugs in the body [17], also based on previous research, the oral bioavailability (OB) and druglikeness (DL) were used as the screening conditions [18]. When OB ≥ 30% and DL ≥ 0.18, the molecule is regarded as a drug that can be absorbed by the human body. The molecular targets were also obtained through TCMSP, and information on each target was entered into UniProt (http://www.uniprot.org/) for correction.

### 2.2. Differentially Expressed Genes Associated with Pneumonia

Differentially expressed genes associated with pneumonia were obtained from NCBI-GEO, a free publicly available microarray database. The GEO accession number is GSE103119 and includes transcriptome data for 152 children with community-acquired pneumonia (CAP) and 20 healthy controls. These data were used only because children are the main population affected by community-acquired pneumonia. Then, these data were analyzed using GEO2R online tools [19], and differentially expressed genes were screened using |logFC| > 1 and an adjusted *P* < 0.05 as indicators.

### 2.3. PPI Network and GO and KEGG Pathway Enrichment Analysis

The ES and SR targets and the abovementioned differential genes are intersected. Then, the protein-protein interaction (PPI) network and Gene Ontology (GO) and Kyoto Encyclopedia of Genes and Genomes (KEGG) pathway enrichment analyses were performed. The PPI network was analyzed using the Search Tool for the Retrieval of Interacting Genes (STRING, http://string.embl.de/) database, the minimum required interaction score is set to medium confidence (0.400), and the organism was *Homo sapiens*. GO enrichment can be divided into 3 main parts including biological process (BP), molecular function (MF), and cellular component (CC). Generally speaking, BP is the main direction of all gene functions in GO [20, 21], and thus, we used GO-BP for enrichment analysis. GO-BP and KEGG pathway enrichment analysis was performed using the clusterProfiler package in R(3.6.1) software, with *p* value cutoff = 0.05 and *q* value cutoff = 0.05 [22] for analysis. Network diagrams were generated with Cytoscape 3.6.0 software.

### 2.4. Chinese Herbs ES and SR

*Ephedra sinica* and *Scutellariae Radix* were purchased from Jiangyin Tianjiang Pharmaceutical Co., Ltd. (Jiangyin, China), and the production batch numbers were 19046614 and 19086524, respectively. These two medicines are marketed prescription drugs approved by the China Food and Drug Administration and are administered as granules.

### 2.5. *Streptococcus pneumoniae* and Mouse Model

*Streptococcus pneumonia*e strain D39 serotype 2 (NCTC 7466) was kindly provided by Professor Jian Huang from Zunyi Medical University (Zunyi, China) and cultured in Todd-Hewitt broth with 1% yeast extract (THY media) at 37°C with 5% CO_2_.

BALB/c mice (female, 6–8 weeks old, 20–22 g) were purchased from the Liaoning Changsheng Biotechnology Co., Ltd. (Shenyang, China) and maintained in accordance with the NIH Guide for the Care and Use of Laboratory Animals. All mouse experiments were approved by the ethics committee of the Changchun University of Chinese Medicine. *Streptococcus pneumoniae* strain D39 was cultured until the OD_600 nm_ reached 0.4 (midlogarithmic phase), collected by centrifugation (1000 rpm for 10 min), and washed three times with PBS. Each mouse was nasally infected with 1.5 × 10^8^ colony-forming units (CFUs) of bacteria, except for those in the healthy control group. The mice were subcutaneously injected with ES (40 mg/kg), SR (40 mg/kg), or DMSO every 8 h, and the lungs from sacrificed mice (*n* = 8) were used for histopathological analysis by hematoxylin-eosin staining under light microscopy at 48 h after infection. Furthermore, other mice were used to collect bronchoalveolar lavage (BAL) fluid, and it was centrifuged. The levels of TNF-*α* and interleukin (IL)-6 were determined in the supernatants using mouse ELISA kits (Biolegend) according to the manufacturer's recommendations.

### 2.6. Molecular Docking

Data for the PLAU (PDB ID: 1C5Z), CD40LG (PDB ID: 3LKJ), BCL2L1 (PDB ID: 3QKD), CASP7 (PDB ID: 1SHJ), CXCL8 (PDB ID: 1QE6), MMP9 (PDB ID: 1GKD), CXCL8 (PDB ID: 1QE6), and MAPK14 (PDB ID: 1A9U) proteins were downloaded from the PDB database (http://www.rcsb.org/). Then, Mgltools 1.5.6 was used to process the protein via hydrogenation, calculate the charge, merge the nonpolar hydrogen, and save it as a pdbqt file. According to the protein's ligand, we defined the active site, set the grid box coordinates, defined the box size as 40 × 40 × 40 grid points, and set the distance of each small grid point as 0.1 nm. AutodockVina 1.1.2 was used for the docking of molecules and proteins. An affinity of less than 0 indicated that the receptor and ligand could spontaneously bind.

### 2.7. Statistical Analysis

Data were expressed by the mean ± SD (*n* ≥ 3) and analyzed by GraphPad Prism 6.0 (GraphPad Software) using the Student's *t*-test; ^*∗∗*^*p* < 0.01.

## 3. Results

### 3.1. ES and SR Can Treat Pneumonia in Mice

**Figure 3:**
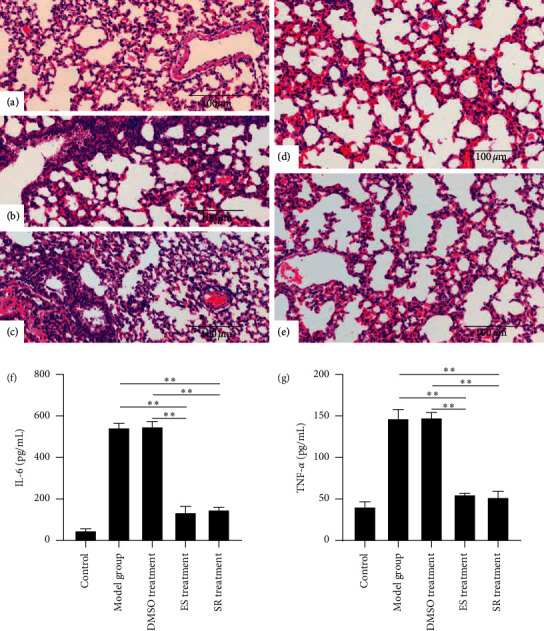
Pathological sections from mice treated with ES and SR. (a) is the control group, (b) is the model group, (c) is the DMSO treatment group, (d) is the ES treatment group, and (e) is the SR treatment group. (f) and (g) are the expression levels of IL-6 and TNF-*α*, respectively. ^*∗∗*^*p* ≤ 0.01.

To evaluate the antimicrobial effect of ES and SR on bacterial pneumonia caused by *Streptococcus pneumoniae*, we conducted a histopathological analysis by hematoxylin-eosin staining in a *Streptococcus pneumoniae*-infected mouse model. As shown in Figure 3, compared with those of the DMSO group, pathological results of the ES and SR groups showed that after ES and SR treatment, exudation and inflammatory cell infiltration were significantly reduced. The levels of IL-6 and TNF-*α* in BAL fluid also indicate the same results. Our results validate the good effects of ES and SR in the treatment of pneumonia.

### 3.2. Physical and Chemical Properties of ES and SR

The results described above drew our attention to the specific mechanisms of ES and SR in the treatment of pneumonia. First, to clearly distinguish the physical and chemical properties of ES and SR, we used 6 specific quantitative indicators in TCMSP data to reflect the molecular characteristics of the drugs: oral bioavailability (OB), druglikeness (DL), molecular weight (MW), the number of donor atoms for H-bonds (nHDon), the number of acceptor atoms for H-bonds (nHAcc), and the Moriguchi octanol–water partition coeff. (log P). The OB and DL represent the pharmacological properties of the drug. As shown in Table 1, ES and SR are very different, and they are completely independent drugs.

### 3.3. Molecular Sets of ES and SR

Furthermore, we screened the molecular sets of the two drugs by OB ≥ 30% and DL ≥ 0.18. ES contains a total of 25 molecules, and SR contains a total of 37 molecules, as shown in Table 2. It is worth noting that although psi-ephedrine and ephedrine have higher OB values, the DL values are only 0.03. However, they are the main alkaloids in ES [23], and these two molecules are even regarded as the main identification indicators of ES in the Chinese Pharmacopoeia. Considering the potential pharmacological effects of these two molecules, we still included these two molecules as the main molecular components of ES. Baicalin has a very high DL value (0.77), but its OB value is only 29.53. Similarly, based on the Chinese Pharmacopoeia and the possible substantial pharmacological effects of this molecule [24], we still regard baicalin as the main molecular component of SR.

### 3.4. Differentially Expressed Genes in Pneumonia

**Figure 4:**
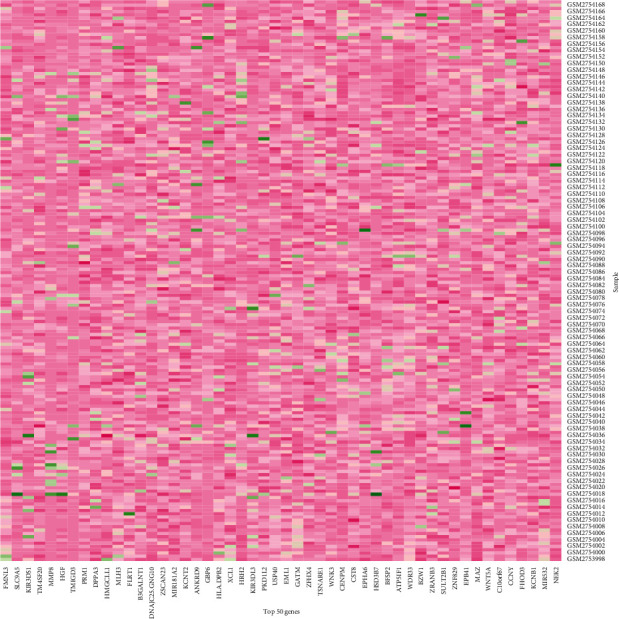
Top 50 differentially expressed genes between healthy and CAP children. Lavender represents high expression, and the green represents low expression.

The differentially expressed genes of 20 normal children and 152 children with CAP are in the supplementary file (Available here). In Figure 4, the first 50 differentially expressed genes are shown.

### 3.5. Active Molecules of ES and SR

**Figure 5:**
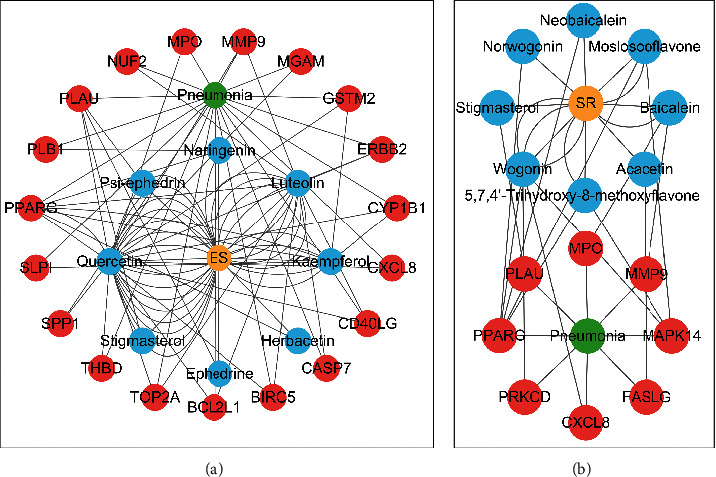
“Drug-Molecule-Target-Disease” network. Yellow represents drugs, blue represents molecules, green represents diseases, and red represents targets.

To further clarify the molecules involved in the interaction between these two drugs and pneumonia, we constructed a “drug-molecule-target-disease” network, as shown in Figure 5. In the ES network, we found that ES contains a total of 8 molecules that can interact with pneumonia-related targets: ephedrine, pseudoephedrine, herbacetin, kaempferol, quercetin, luteolin, stigmasterol, and naringenin. As mentioned earlier, ephedrine and psi-ephedrine, sympathetic amines with unique effects and an indirect mechanism of action, have a unique effect among sympathetic drugs, such as phenylephrine [25]; they are the two main components of ES and are usually used to constrict blood vessels [26] and treat colds [27]. Herbacetin is a natural flavonoid [28] and has been shown to have antioxidant, anticancer, and anti-inflammatory effects [29]. Kaempferol also has anticancer [30] and anti-inflammatory [31] effects. Quercetin has many functions, including regulating blood pressure [32], mediating anticancer effects [33], and treating inflammation and pain [34]. Luteolin has also been reported to have good anti-inflammatory effects [35]. Stigmasterol, a phytosterol, has cholesterol-lowering activity [36] and is valued as an anti-osteoarthritic agent [37]. Naringenin is a potential immunomodulator in therapeutics [38].

Coincidentally, in the SR network, we also found that eight molecules played a major role in the alleviation of pneumonia. They are wogonin, acacetin, baicalein, 5,7,4′-trihydroxy-8-methoxyflavone, neobaicalein, norwogonin, stigmasterol, and moslosooflavone. Baicalein is the main compound of SR. It inhibits apoptosis and autophagy [39] and protects nerves [40]. Wogonin has strong antitumor activity [41]. Acacetin has antioxidative and anti-inflammatory effects [42] as well as protective effects on cerebral blood vessels [43]. 5,7,4′-Trihydroxy-8-methoxyflavone can be used to treat influenza and its potential complications [44]. Neobaicalein has anticancer effects [45]. Norwogonin has antibacterial [46] and antidiabetic [47] activity. Moslosooflavone may have antihypoxia activity [48] and antivirus [49] effects. It is worth noting that stigmasterol is present in both ES and SR, and the intersection of this active ingredient may suggest that ES and SR also have a synergistic effect to some extent. The above ES and SR ingredients are flavonoids, except for stigmasterol, ephedrine, and psi-ephedrine. It has been proven that the main active molecules of ES and SR are mostly anti-inflammatory, antibacterial, or antiviral. However, we believe that ES and SR may act on different pathways.

### 3.6. GO-BP and KEGG Enrichment Analysis

**Figure 6:**
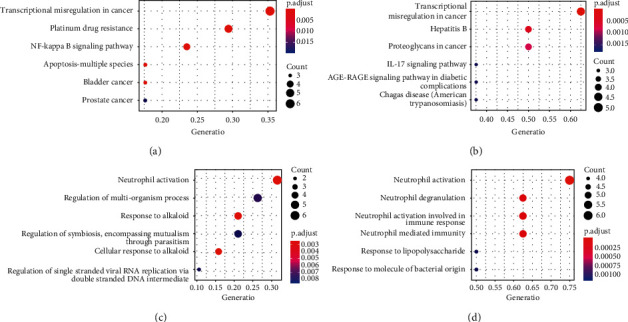
GO-BP and KEGG enrichment results for ES and SR. (a) KEGG enrichment result for ES, (b) KEGG enrichment result for SR, (c) GO-BP enrichment result for ES, and (d) GO-BP enrichment result for SR.

In addition to the drug components, the above research also provided information on 19 *Ephedra* and 8 *Scutellaria* targets in pneumonia. To further explain the biological functions corresponding to the targets, GO-BP and KEGG functions were used to enrich them. The results are shown in Figure 6. The GO-BP enrichment results suggest that ES can regulate neutrophil activation, the multiorganism process, the response to alkaloids, and others. Additionally, SR can regulate neutrophil activation, neutrophil degranulation, neutrophil activation involved in the immune response, and others. Regardless of the disease pathway, the KEGG enrichment results suggest that ES plays a therapeutic role through the NF-*κ*B and apoptosis signaling pathways, while SR acts mainly through the IL-17 signaling pathway.

NF-*κ*B is considered to be a central regulator of the inflammatory process and an important participant in innate and adaptive immune responses [50]. It also plays a key role in regulating the expression of hundreds of immune-related genes, especially those encoding proinflammatory factors, chemokines, and other genes important for the development of the immune system [51]. Apoptosis usually occurs during development and aging and is an in vivo homeostasis mechanism for maintaining the number of cells in tissue [52]. The dysfunction of apoptosis leads to the release of a large number of proinflammatory factors, such as IL-1, IL-6, and IL-12, and it increases the immune burden on phagocytic cells. Excessive apoptosis of alveolar epithelial cells is an important mechanism of lung injury [53]. Therefore, it is necessary to maintain apoptosis in a dynamic balance and control excessive and abnormal apoptosis [54].

As a characteristic cytokine secreted by TH17 cells, IL-17 plays an important role in autoimmune diseases and immune-inflammatory damage diseases. There are 6 cytokines in the IL-17 family: IL-17A, IL-17B, IL-17C, IL-17D, IL-17E (IL-25), and IL-17F. Among them, IL-17A has been the most studied and is usually directly referred to as IL-17 [55]; it can induce a variety of proinflammatory factors, including IL-6, IL-1*β*, and TNF-*α* [56]. In particular, IL-6 can further promote the differentiation and activation of TH17 cells through positive feedback, forming a cytokine storm and aggravating inflammation [57]. We aimed to verify how ES and SR work on these three pathways.

### 3.7. PPI Network

**Figure 7:**
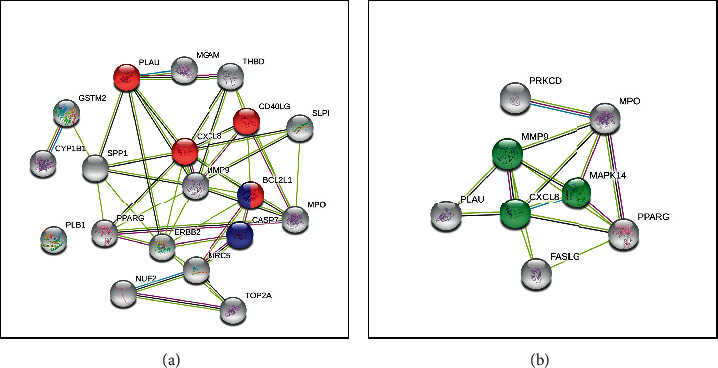
PPI networks of ES and SR. (a) The network for ES; red represents proteins related to the NF-*κ*B pathway, and blue represents proteins related to apoptosis. (b) The networks for SR; green represents proteins related to the IL-17 signaling pathway.

As the executors of cellular activities and functions, proteins do not exist independently. The interactions between proteins play an important role in each stage of life and maintain the steady state of the internal environment. To analyze the interaction between ES and SR target proteins, a PPI network of target proteins was constructed, as shown in Figure 7. We labeled NF-*κ*B-related, apoptosis-related, and IL-17-related proteins in the figure for further verification.

### 3.8. Molecular Docking

**Figure 8:**
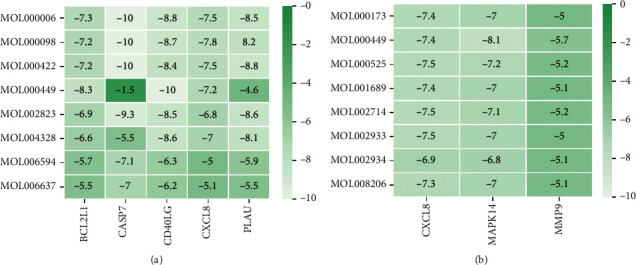
Molecular docking of ES and SR to the targets. (a) ES and (b) SR. The numbers in the table represent the affinity, and an affinity less than 0 indicates successful docking.

Above, we proved that the 8 active ingredients of ES may play a role in treating pneumonia by targeting the NF-*κ*B pathway and the apoptosis pathway, and the 8 active ingredients of SR may play a role in treating pneumonia by targeting the IL-17 signaling pathway. However, these data were not sufficient to verify our results. We used the main target proteins of the NF-*κ*B pathway, apoptosis pathway, and IL-17 signaling pathway in the PPI network, including PLAU, CD40LG, BLC2L1, CASP7, MMP9, CXCL8, and MAPK14, for verification. As shown in Figure 8, all components could be successfully docked with the target protein. In conclusion, our study proved that LAU, CD40LG, BLC2L1, CASP7, MMP9, CXCL8, and MAPK14 are the main targets of ES and SR.

## 4. Discussion

Pneumonia is a serious global health problem. Antibiotics, vaccines, bacteriophages, and antimicrobial peptides are all considered to be treatments for bacterial pneumonia, but they are not perfect [58]. Chinese medicine is a traditional therapy that has been used in China for thousands of years and has the characteristics of multisystem and multitarget treatment. Due to the incomplete knowledge, research on traditional Chinese medicine is limited to the molecules contained in medicinal materials. It is believed that traditional Chinese medicinal materials are collections of multiple molecules. There are few studies on the mechanisms and targets of traditional Chinese medicines used for the treatment of pneumonia.

In this study, we first put forward the theory of treating the same disease in different ways in traditional Chinese medicine. To prove this theory and investigate the compound structures of ES and SR in more detail, we used a database constructed by previous researchers [16] and obtained differentially expressed genes in patients with pneumonia from the GEO database. Based on the network pharmacology method, the specific mechanisms of the two traditional Chinese medicines in the treatment of pneumonia were studied to clarify the four types of qi in Chinese medicine and guide clinical treatment.

In animal experiments, the alleviation of the effects of ES and SR, two traditional Chinese medicines, on pneumonia was proven. Then, we used OB ≥ 30% and DL ≥ 0.18 to thoroughly investigate the specific molecular components of ES and SR. Through the GEO database, information on differentially expressed genes associated with pneumonia was found. Based on the intersections of molecular target genes and differentially expressed genes associated with pneumonia, we believe that ES and SR have 8 effective components that can act on pneumonia. After GO-BP and KEGG enrichment of related genes, we found that ES can treat pneumonia through NF-*κ*B and apoptosis and ES can play a therapeutic role through the IL-17 signaling pathway.

**Figure 9:**
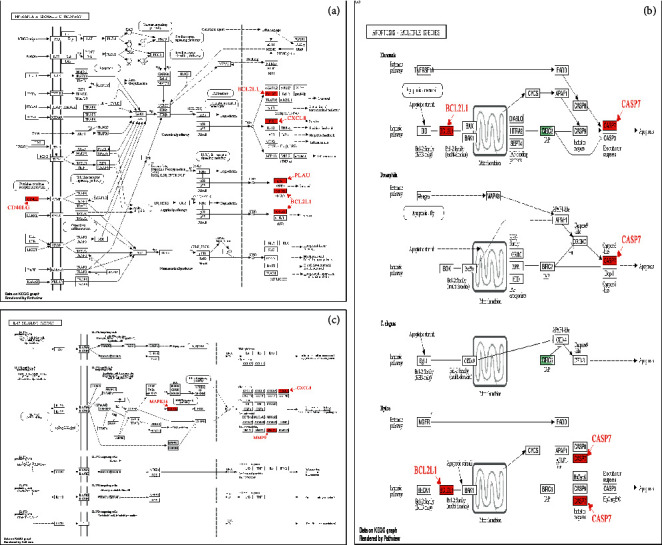
Pathways of ES and SR. (a) is the NF-*κ*B signaling pathway, (b) is the apoptosis signaling pathway, and (c) is the IL-17 signaling pathway. Red is the targets of ES and SR.

Furthermore, we used molecular docking to verify the results and found that ES can regulate the NF-*κ*B signaling pathway-related proteins PLAU, CD40LG, BLC2L1, and CXCL8. In addition, it can regulate the BLC2L1 (also related to NF-*κ*B) and CASP7 proteins related to the apoptosis pathway. SR can regulate the MMP9, CXCL8, and MAPK14 proteins related to the IL-17 signaling pathway. In summary, we reached the following conclusions:

1. ES and SR can both treat pneumonia in vivo.
2. The active ingredients of ES for pneumonia are ephedrine, psi-ephedrine, herbacetin, kaempferol, quercetin, luteolin, stigmasterol, and naringenin.
3. The active ingredients of SR for pneumonia are wogonin, acacetin, baicalein, 5,7,4′-trihydroxy-8-methoxyflavone, neobaicalein, norwogonin, stigmasterol, and moslosooflavone.
4. ES plays a therapeutic role through the NF-*κ*B and apoptosis signaling pathways, and SR plays a therapeutic role through the IL-17 signaling pathway.
5. The eight main molecules of ES exerted a marked effect in treating pneumonia by targeting the NF-*κ*B and apoptosis pathways through the PLAU, CD40LG, BLC2L1, CASP7, and CXCL8 proteins. The eight main components of SR can treat pneumonia by targeting the IL-17 signaling pathway though the CXCL8, MAPK14, and MMP9 proteins, as shown in Figure 9.
6. Most importantly, ES, as a representative warm medicine, and SR, as a representative cold medicine, can both treat pneumonia.

**Figure 10:**
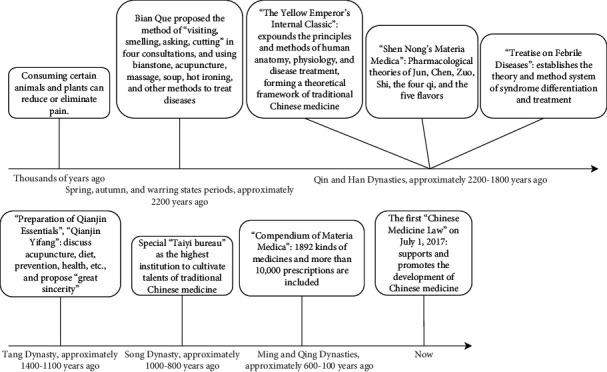
History of TCM development.

From another perspective, our ancestors found that in food preparation, labor, and the struggle with nature, they could use animal skins, bark bags, hot stones, or sandy soil as part of the heat for heating. Heating could eliminate some pain and gradually lead to the development of hot ironing and moxibustion. When using stone tools to work, it was found that after puncturing one part of the body, the pain in other parts of the body could be relieved, thereby creating a method of treating pain with vermiculite and bone needles. The consumption of certain animals and plants has the effect of reducing or eliminating disease, which is the origin of Chinese medicine. As humans evolved, they began to purposefully search for drugs and methods to prevent and treat diseases and developed primitive Chinese medicine. Later, with further development, traditional Chinese medicine was continuously improved by the Chinese people in terms of theory and methods. As shown in Figure 10, these developments gradually led to the current medicines and system. However, the holistic concept holds that human beings, nature, and society are a whole. Dialectical treatment emphasizes individualized treatment for each person. It also emphasizes the prevention-oriented concept of treating the disease and holds that disease resistance can be improved through emotional adjustment, moderate work and rest, a reasonable diet, and regular routines to achieve health and disease prevention. The four qi theory of Chinese medicine in this study belongs to the dialectical governance theory. These theories and practices greatly protected the health of ancient Chinese people and continue to protect health to this day. There is no doubt that most of these theories are related to philosophy, and only the Chinese people, with the cultural heritage passed down from their ancestors, can understand these ideas without further learning. To date, traditional Chinese medicine has been regarded as a complementary and alternative medicine, and the data support this view. However, looking at the history of modern medicine, from bloodletting therapy to the use of antibiotics and the sequencing of the human genome, the degree of human understanding of the disease is closely related to developments in the understanding of the human body. The development of modern medicine is a history of scientific and technological progress. Modern medicine continues to push the limits of technology. Organ, tissue, and cell transplants, test-tube babies, laser medicine, and other developments have all contributed to protecting human health. Modern medicine is undoubtedly mainstream medicine in modern society. However, we should not forget the traditional medicine that has treated countless people just because better treatments are available. Modern medicine also has many unsolved problems, such as AIDS, Alzheimer's disease, and even diabetes. No one can guarantee that traditional Chinese medicine will not make some breakthroughs in these unsolved fields, similar to Tu [59]. The potential utility of Chinese herbs in clinical practice in the future includes replacing or reducing the use of antibiotics [60, 61], reducing the overuse of hormone drugs [62], antivirus activity [63], antitumor activity [64], improving immunity [65], and others. After all, humans know too little about the world.

In short, in this study, we take ES and SR as examples and use a combination of network pharmacology and transcriptomics and experimental results to prove the TCM theory of treating the same disease with different treatments. We think it is very important to explore the theory and continue the practice of TCM.

## Figures and Tables

**Table 1:** Physicochemical properties of *Ephedra* and *Scutellaria*.

Herb	*n*	MW	nHDon	nHAcc	log P	OB	DL
ES	363	202.72 (100.40)	0.85 (1.51)	1.81 (2.28)	3.47 (2.72)	37.12 (19.11)	0.11 (0.17)
SR	143	277.73 (106.36)	1.44 (1.92)	3.47 (3.21)	4.10 (3.33)	31.41 (18.67)	0.23 (0.21)

The values in the table are the mean (SD).

**Table 2:** Molecular sets of ES and SR after screening.

Molid	Compound	OB	DL	Structure
ES1 MOL010788	Leucopelargonidin	57.97	0.24	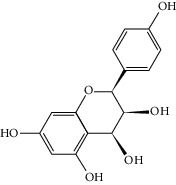
ES2 MOL002823	Herbacetin	36.07	0.27	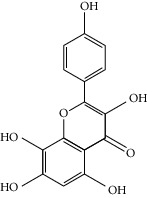
ES3 MOL010489	Resivit	30.84	0.27	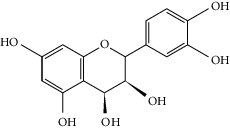
ES4 MOL000422	Kaempferol	41.88	0.24	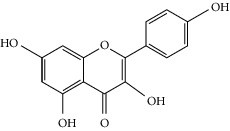
ES5 MOL004798	Delphinidin	40.63	0.28	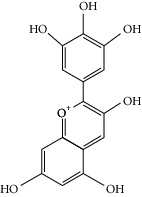
ES6 MOL000098	Quercetin	46.43	0.28	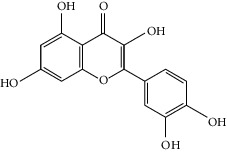
ES7 MOL000006	Luteolin	36.16	0.25	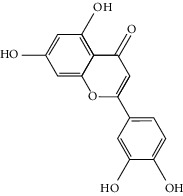
ES8 MOL000358	Beta-sitosterol	36.91	0.75	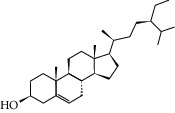
ES9 MOL000449	Stigmasterol	43.83	0.76	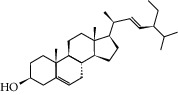
ES10 MOL000492	(+)-catechin	54.83	0.24	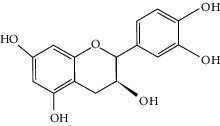
ES11 MOL001494	Mandenol	42	0.19	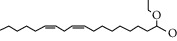
ES12 MOL001506	Supraene	33.55	0.42	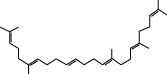
ES13 MOL001755	24-Ethylcholest-4-en-3-one	36.08	0.76	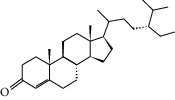
ES14 MOL001771	Poriferast-5-en-3beta-ol	36.91	0.75	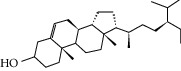
ES15 MOL002881	Diosmetin	31.14	0.27	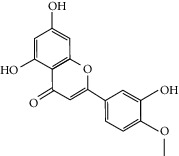
ES16 MOL004328	Naringenin	59.29	0.21	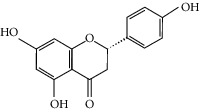
ES17 MOL004576	Taxifolin	57.84	0.27	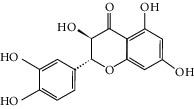
ES18 MOL005043	Campest-5-en-3beta-ol	37.58	0.71	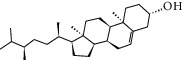
ES19 MOL005190	Eriodictyol	71.79	0.24	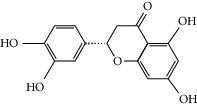
ES20 MOL005573	Genkwanin	37.13	0.24	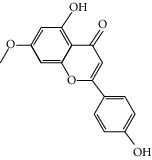
ES21 MOL005842	Pectolinarigenin	41.17	0.3	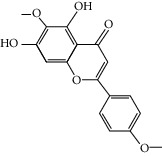
ES22 MOL007214	(+)-Leucocyanidin	37.61	0.27	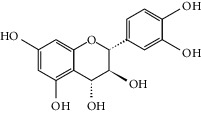
ES23 MOL011319	Truflex OBP	43.74	0.24	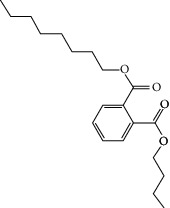
ES24 MOL006637	Psi-ephedrine	52.25	0.03	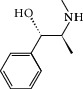
ES25 MOL006594	Ephedrine	43.35	0.03	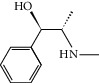
SR1 MOL001689	Acacetin	34.97	0.24	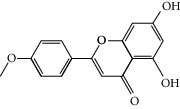
SR2 MOL000173	Wogonin	30.68	0.23	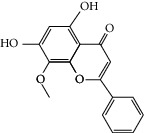
SR3 MOL000228	(2R)-7-Hydroxy-5-methoxy-2-phenylchroman-4-one	55.23	0.2	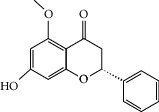
SR4 MOL002714	Baicalein	33.52	0.21	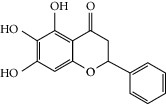
SR5 MOL002908	5,8,2′-Trihydroxy-7-methoxyflavone	37.01	0.27	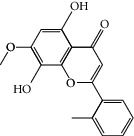
SR6 MOL002909	5,7,2,5-Tetrahydroxy-8,6-dimethoxyflavone	33.82	0.45	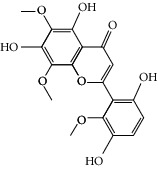
SR7 MOL002910	Carthamidin	41.15	0.24	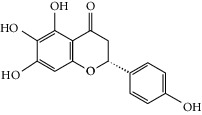
SR8 MOL002911	2,6,2′,4′-Tetrahydroxy-6′-methoxychaleone	69.04	0.22	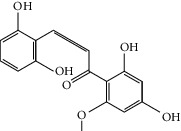
SR9 MOL002913	Dihydrobaicalin_qt	40.04	0.21	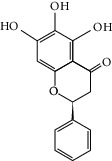
SR10 MOL002914	Eriodyctiol (flavanone)	41.35	0.24	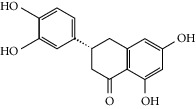
SR11 MOL002915	Salvigenin	49.07	0.33	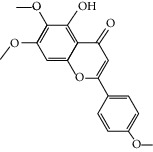
SR12 MOL002917	5,2′,6′-Trihydroxy-7,8-dimethoxyflavone	45.05	0.33	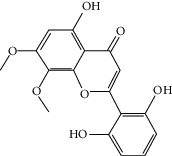
SR13 MOL002925	5,7,2′,6′-Tetrahydroxyflavone	37.01	0.24	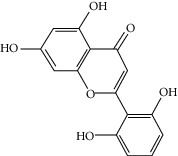
SR14 MOL002926	Dihydrooroxylin A	38.72	0.23	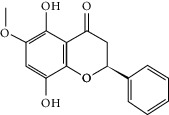
SR15 MOL002927	Skullcapflavone II	69.51	0.44	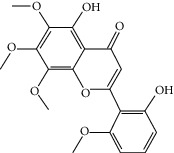
SR16 MOL002928	Oroxylin a	41.37	0.23	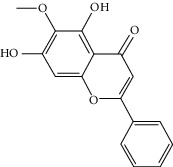
SR17 MOL002932	Panicolin	76.26	0.29	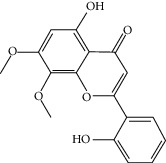
SR18 MOL002933	5,7,4′-Trihydroxy-8-methoxyflavone	36.56	0.27	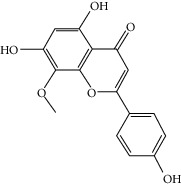
SR19 MOL002934	Neobaicalein	104.34	0.44	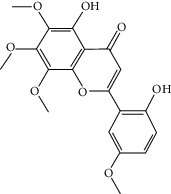
SR20 MOL002937	Dihydrooroxylin	66.06	0.23	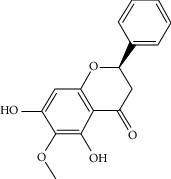
SR21 MOL000358	Beta-sitosterol	36.91	0.75	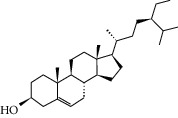
SR22 MOL000359	Sitosterol	36.91	0.75	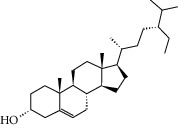
SR23 MOL000525	Norwogonin	39.4	0.21	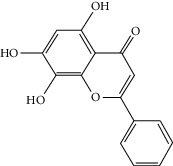
SR24 MOL000552	5,2′-Dihydroxy-6,7,8-trimethoxyflavone	31.71	0.35	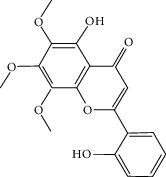
SR25 MOL000073	Ent-epicatechin	48.96	0.24	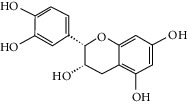
SR26 MOL000449	Stigmasterol	43.83	0.76	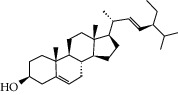
SR27 MOL001458	Coptisine	30.67	0.86	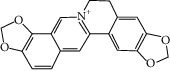
SR28 MOL001490	Bis [(2S)-2-ethylhexyl] benzene-1,2-dicarboxylate	43.59	0.35	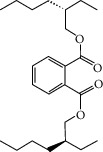
SR29 MOL001506	Supraene	33.55	0.42	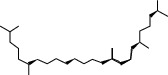
SR30 MOL002879	Diop	43.59	0.39	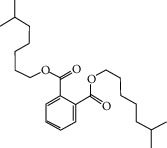
SR31 MOL002897	Epiberberine	43.09	0.78	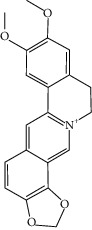
SR32 MOL008206	Moslosooflavone	44.09	0.25	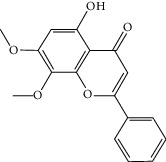
SR33 MOL010415	11,13-Eicosadienoic acid, methyl ester	39.28	0.23	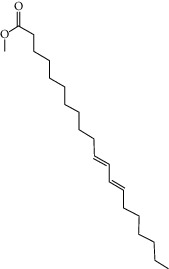
SR34 MOL012245	5,7,4′-Trihydroxy-6-methoxyflavanone	36.63	0.27	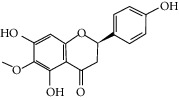
SR35 MOL012246	5,7,4′-Trihydroxy-8-methoxyflavanone	74.24	0.26	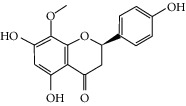
SR36 MOL012266	Rivularin	37.94	0.37	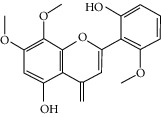
SR37 MOL002935	Baicalin	29.53	0.77	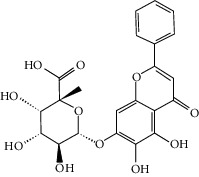

## Data Availability

The data used to support the findings of this study are included within the supplementary information file.

## Supplementary Materials

Supplementary Materials The differentially expressed genes of 20 normal children and 152 children with CAP.
